# BoCaTFBS: a boosted cascade learner to refine the binding sites suggested by ChIP-chip experiments

**DOI:** 10.1186/gb-2006-7-11-r102

**Published:** 2006-11-01

**Authors:** Lu-yong Wang, Michael Snyder, Mark Gerstein

**Affiliations:** 1 Integrated Data Systems Department, Siemens Corporate Research, 755 College Road East, Princeton, New Jersey 08540, USA; 2 Department of Molecular, Cellular, and Developmental Biology, KBT 926, 266 Whitney Ave, Yale University, New Haven, Connecticut 06520, USA; 3 Department of Molecular Biophysics and Biochemistry, Bass 432A, 266 Whitney Ave, Yale University, New Haven, CT 06520, USA; 4 Program in Computational Biology and Bioinformatics, Bass 432A, 266 Whitney Ave, Yale University, New Haven, CT 06520, USA; 5 Department of Computer Science, 51 Prospect Street, Yale University, New Haven, Connecticut 06520, USA

## Abstract

Comprehensive mapping of transcription factor binding sites is essential in postgenomic biology. For this, we propose a mining approach combining noisy data from ChIP (chromatin immunoprecipitation)-chip experiments with known binding site patterns. Our method (BoCaTFBS) uses boosted cascades of classifiers for optimum efficiency, in which components are alternating decision trees; it exploits interpositional correlations; and it explicitly integrates massive negative information from ChIP-chip experiments. We applied BoCaTFBS within the ENCODE project and showed that it outperforms many traditional binding site identification methods (for instance, profiles).

## Abstract 2

BoCaTFBS, a new method that combines noisy data from ChIP-chip experiments with known binding-site patterns, is described and applied to the ENCODE project.

## Background

The diverse phenotypes from an invariant set of genes are controlled by a biochemical process that regulates gene activity [1]. Transcription is central to the regulation mechanisms in the process of gene expression. It is regulated by interplay between transcription factors and their binding sites.

Understanding the targets that are regulated by transcription factors in the human genome is highly desirable in the postgenomic era. Some experimental methods, such as footprinting [2] and SELEX (systematic evolution of ligands by exponential evolution) [3], exist for identifying transcription factor binding sites (TFBSs). Chromatin immunoprecipitation (ChIP)-chip technology was introduced originally to identify genomic binding regions of transcription factors in yeast [4-6]. It was later applied to the human genome [7]. There have been many applications to single chromosomes in human. ChIP-chip technology, otherwise known as microarray-based readout of chromatin immunoprecipitation assays, is a procedure for mapping *in vivo *targets of transcription factors by ChIP with antibodies to a transcription factor of interest in order to isolate protein-bound DNA, followed by probing a microarray containing genomic DNA sequences with the immunoprecipitated DNA.

Snyder and colleagues [8] mapped nuclear factor (NF)-κB binding sites in human chromosome 22 in a high-throughput manner. A number of other publications have similarly mapped the sites of other transcription factors [9,10]. ChIP-chip technology has been applied to the human genome for a variety of different factors [11]. Additionally, there are related techniques such as ChIP-SAGE (serial analysis of gene expression) [12-14]. Unfortunately, the ChIP-chip technique and its variants are still time consuming, sensitive to the physiologic perturbation, and expensive to use for screening TFBSs in the whole genome.

Many computational methods for identifying TFBSs have been proposed in the literature [15-17]. Some of the methods attempt to discover potential binding sites for any transcription factor given only a collection of unaligned promoter regions for suspected coregulated genes (for example MEME [18], AlignAce [Gibbs sampling] [19], and BioProspector [20]). Other methods attempt to predict TFBSs for a specific transcription factor given a collection of known binding sites already available [15,21-23]. Our proposed method in this paper is relevant to the latter problem.

Consensus sequences or regular expressions are still frequently used to depict the binding specificities of transcription factors. They represent a somewhat simplistic view of the binding sequence and only work well in highly conserved motifs because they do not contain useful information about the relative likelihood of observing the alternate nucleotides at different positions of a TFBS. However, variability is believed to have a critical impact on the fine regulation of gene expression. This makes it very difficult to identify all potential binding sites without the aid of computational techniques.

Another more common method is the profile method, also known as positional specific scoring matrix (PSSM) or position weight matrix [21]. The largest and most commonly used collection is the TRANSFAC database, which catalogs transcription factors, their known binding sites, and the corresponding profiles (PSSMs) [23]. In addition, a number of tools such as MATRIX SEARCH [24], MatInd/MatInspector [25], Mapper [26], SIGNAL SCAN [27], and rVISTA [28], have been developed to enable the user to search an input sequence for matches to a PSSM or a library of PSSMs. However, PSSMs treat each position of the binding sites as independent from each other. They cannot model the interactions between positions within DNA-binding sites, nor can they model explicit coevolution of related positions within binding sites. PSSMs normally describe only a fixed length motif, whereas many DNA-binding proteins can bind to variable length sites. Finally, it is not always feasible to construct a multiple alignment of the binding sites necessary to build a PSSM.

Graphical models were also introduced to represent the dependences between positions [29,30]. In particular, Markov chains were utilized to statistically model the number and relative locations of TFBSs within a sequence. Although the hidden Markov model allows dependencies among positions to be encoded in the state transition probabilities [29], not all dependencies are well treated systematically. An optimized Markov chain algorithm was introduced to integrate pair-wise correlation into Markov models to predict a particular transcription factor's binding sites (hepatocyte nuclear factor 4α) [22].

An alternative approach, phylogenetic footprinting, identifies functional regulation elements from noncoding DNA sequence conservation between related species [31-33]. It has successfully been applied to single genome loci, but this method is limited by the short length of functional binding sites and the large number of insertion/deletion events within regulatory regions. There are also other methods, such as maximal dependence decomposition [34] and the nonparametric method [35]. Singh and coworkers [15] evaluated traditional TFBS prediction methods and introduced per-position information content and local pair-wise nucleotide dependencies to four major traditional methods (for further detail, see Materials and methods, below). Their benchmark results on *Escherichia coli *transcription factors indicated that the best results were achieved by incorporating both per-position information content and local pair-wise correlation; however, all of the conventional methods of TFBS prediction generate a high false-positive rate when applied to the genome [36].

Local pair-wise correlation within TFBSs was discovered in some recent experimental and theoretical research. Microarray binding experiments indicated that nucleotides of TFBSs exert interdependent effects on the binding affinities of transcription factors [37]. Also, Kwiatkowski and coworkers [38] showed that there are nucleotide positions in the TFBSs that interact with each other by using principle coordinate analysis to predict the effects of single nucleotide polymorphisms within regulatory sequences on DNA-protein interactions.

Finding TFBSs is particularly challenging in the human genome in comparison with simpler organisms such as yeast and fly. TFBSs can occur downstream, upstream, or possibly in the introns of the genes they regulate [8-10]. Moreover, the human genome is about 200 times larger than the yeast genome, and approximately 99% does not encode proteins. Thus, it can be very difficult to find TFBSs in noncoding sequences using relatively simple computational tools.

In this postgenomic era, comprehensive high-throughput experiments (such as ChIP-chip) or gene annotation provides a huge amount of information about sites that are not bound by a factor, as well as some information about the sites that are bound. In fact, such techniques provide better information about nonbinding sites than about binding sites because the resolution of the binding sites is limited by the size of probes in the ChIP-chip experiments and there are only limited binding regions detected, whereas there is a very large amount of information on sites not bound. Moreover, the ENCyclopedia Of DNA Elements (ENCODE) Project [39] is expected to produce a surge in the availability of massive ChIP-chip datasets.

Here we propose a general and robust method for automatically identifying TFBSs. Because an enormous amount of nonbinding information has been generated from ChIP-chip experiments, our new method should not only be able to utilize positional information and interpositional correlation in TFBSs, but it should also systematically incorporate information from the numerous nonbinding sites.

Our method is designed to harness specifically this information about sites that are not bound. We call this negative information 'massive nonbinding site information'. The nonbinding regions from yeast were recently used in another computational method proposed by Hong and coworkers. In particular, those investigators described a single boosting approach (MotifBooster) and applied it to yeast ChIP-chip data [40]. MotifBooster classifies the bound and nonbound regions of ChIP-chip experiments, and represents a significant innovation by explicitly including the nonbinding region information. A single boosting classifier using PSSMs as the basis for its weak classifiers was trained over the yeast ChIP-chip datasets. However, in the human genome, data become substantially more massive and the distribution of the class labels (binding or nonbinding) is even more skewed. As is described below, to train a single boosting classifier can be difficult for the whole human genome because of the computational inefficiency for training over massive datasets [41].

Efficiency and scalability are key challenges for handling massive datasets in a boosting paradigm [42]. The amount of nonbinding information in the whole human genome ChIP-chip experiment is truly massive [39]. It is on the order of billions (3 million negative probes multiplied by their average length of 1000 base pairs [bp]). It is critical to incorporate efficiently the large scale negative, nonbinding information. One of the issues for a standard boosting method is that it must consider sequentially all of the positive and negative instances at each iteration of the boosting process. However, when the size of the dataset becomes very large, efficiency and scalability issues arise. A straightforward static sampling over such a large dataset may result in a significant loss of information and a potentially biased classifier. A standard boosting algorithm can not deal with such datasets efficiently [42].

In this report we propose an efficient and effective classification method based on a boosted cascade of ADTboost in order to predict the TFBSs, focusing on the human genome. Our method (which we call 'BoCaTFBS') is specifically designed to be coupled with ChIP-chip experiments. These experiments only give an approximation of the locations of binding regions, but they produce a massive amount of nonbinding information. We use this massive nonbinding information and the known binding information for prediction of the binding sites. Our method efficiently integrates nonbinding information as well as positional information and interpositional relationships. Thus, it has many advantages in identifying TFBSs. First, we trained BoCaTFBS with negative samples in addition to positive samples in order to decrease the high positive rate inherent in traditional methods such as PSSM. Second, its efficient cascade structure quickly discards the 'easy' over-represented class samples and focuses on the 'harder' ones and the promising regions. This boosted cascade procedure improves the detection performance through stages and decreases the computation time, which is an important consideration for genome-scale applications. Third, there is massive nonbinding site information and only limited binding site information. Thus, classification may be biased toward the over-represented class. The boosted cascade also solves the imbalance issue by random subset selection and removal of the over-represented set in an inherent, natural way. Fourth, the BoCaTFBS method uses ADTboost as the learner for each stage. It considers features from both positions and relationships among positions within TFBSs. ADTboost provides classification with a real-valued measurement, whose absolute value has been interpreted as a confidence measure. One of the features of ADTboost is that it generates classification rules that are smaller and easier to interpret than other machine learning methods (such as support vector machine and neural networks).

In addition to presenting this method, we benchmarked performance of *BoCaTFBS*. We comprehensively compared it with many traditional methods (PSSM, Centroid, Berg von Hippel, consensus, and their improved variants), 'crippled' BoCaTFBS, and single boosting algorithm. Moreover, we applied BoCaTFBS to ongoing ENCODE projects.

## Results

### Cross-validation and receiver operating characteristic analysis

At first, experimental results of NF-κB binding sites in human chromosome 22 were utilized to benchmark our method. Repetitive 10-fold cross-validation was performed for our BoCaTFBS method (see Materials and methods, below), as well as for four traditional methods in TFBS prediction: consensus, PSSM, Berg and von Hippel (BvH), and centroid.

**Figure 1:**
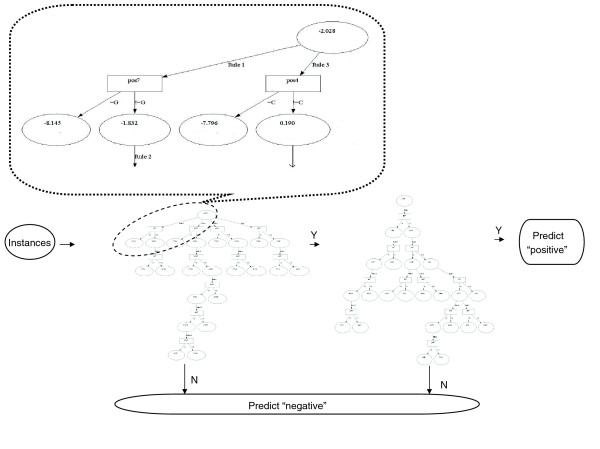
A BoCaTFBS classifier trained over NF-κB ChIP-chip experimental data. It consists of two cascade stages and 12 features for each stage (partially shown). This cascade predictor was tested by cross-validation and achieved 82% sensitivity (true positive rate) at a 5% false-positive rate. BoCaTFBS classifiers are built on discriminative features, which differentiate positives (the binding sites) from the chosen negative training set (the nonbinding sites). For example, in stage 1, the sequence where position 4 is not C is more likely to have more binding propensity. The consensus sequence of binding sites is GGGRNNYYCC (R is purine, Y is pyrimidine, and N is any nucleotide). The classifier at each stage is built upon a random small subset of the over-represented class at each stage. Moreover, each classifier is dependent on the results of the classifiers in the previous stages. NF-κB, nuclear factor-κB.

In principle, one could define an optimization framework in which the number of classifier stages and the number of boosting steps in each stage are traded off during the cascade training. Unfortunately, finding this optimum is a difficult and impractical problem [41,43]. In practice, a very simple approach is used to produce an effective classifier empirically. An arbitrary number of cascade stages and number of boosting steps in each stage may be predefined. These parameters are adjusted and determined by testing on a randomly selected small validation subset for good performance. The boosting procedure will stop if adding one more base classifier or cascade stage increases the error for the reserved validation set. An example is shown in Figure 1. Two cascade stages and 12 features in each stage are predefined for NF-κB binding site prediction. This cascade predictor was tested by cross-validation, and shows 82% sensitivity (true positive rate) at a 5% false-positive rate. The resulting classifier incorporates discriminative features, rather than just the descriptive features, and differentiates the binding sites from the nonbinding sites. In contrast, the single ADTboost classifier at the first cascade stage shows 71% true positive rate at 5% false positive rate. It seems that the further stage refines the positive prediction and increases the true positive rate over the prior cascade stages.

**Figure 2:**
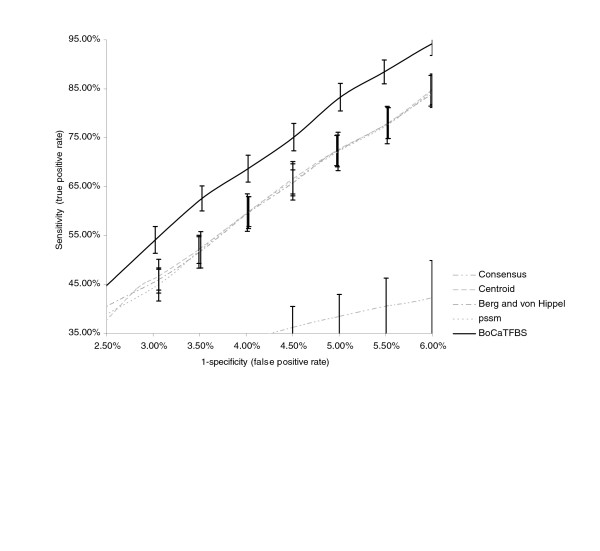
ROC curves depicting the performance of BoCaTFBS versus that of traditional methods. The traditional methods considered included centroid, Berg and von Hippel, PSSM, and consensus. False positive rate, also known as 1-specificity, is defined as the ratio of false positives over the sum of false positives and true negatives. True positive rate, also known as sensitivity, is defined as the ratio of true positives over the sum of true positives and false negatives. The error bars are 95% confidence intervals. Our *BoCaTFBS *method notably outperforms the other four methods. PSSM, positional specific scoring matrix; ROC, receiver operating characteristic.

Figure 2 shows the receiver operating characteristic (ROC) curve analysis results based on the performance of these five methods. Each ROC curve plots the percentage of correctly predicted positive examples (true positive rate; specifically, the ratio of true positives over the sum of true positives and false negatives) as a function of the percentage of incorrectly predicted negative examples (false positive rate; namely the ratio of the false positives over the sum of false positives and true negatives).

The results indicate that our BoCaTFBS method performs consistently better than all four traditional methods. For example, at the 5.5% false-positive rate level, the sensitivity of our method is approximately 11% higher than the centroid, BvH, and PSSM approaches. At each specificity level, the true positive rate of our BoCaTFBS prediction method is clearly higher than the other methods, whereas the false-positive rate of our method is less than that with the other methods at each sensitivity level. The consensus approach has the worst performance, as anticipated; the other three traditional methods had comparable performance. Additionally, for our *BoCaTFBS *method, a *P *value was estimated by permuting the dataset labels ('binding' or 'nonbinding') randomly and re-evaluating the sensitivity rate at the same specificity level (5.5%). We permuted the dataset 1000 times and found that none of the classifiers had better sensitivity at the same specificity level. This shows empirically that the *P *value is less than 1/1000.

### Comparison with positional information methods

We compared our BoCaTFBS method with the improved methods reported by Singh and coworkers [15], which introduced the per-position information content and pair-wise correlations with the four traditional methods (described in Materials and methods, below). Cross-validation and comparative studies were performed between these methods and our BoCaTFBS method on NF-κB binding prediction by ROC analysis.

**Figure 3:**
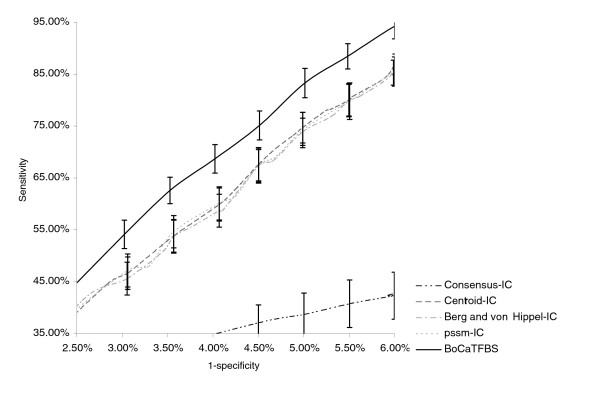
ROC curves comparing BoCaTFBS with centroid-IC, BvH-IC, PSSM-IC, and consensus-IC methods. The latter four methods are the four traditional methods incorporating per-position IC [15]. The error bars are 95% confidence intervals. Our BoCaTFBS method clearly outperforms the other four methods. BvH, Berg and von Hippel; IC, information content; PSSM, positional specific scoring matrix; ROC, receiver operating characteristic.

Figure 3 evaluated the performance of our BoCaTFBS method and the other four methods incorporating the per-position information content (IC). The results indicate that our BoCaTFBS method consistently outperforms the other four methods utilizing the per-position IC. At the 5.5% false-positive rate level, for example, our boosted cascade method outperforms the centroid-IC, BvH-IC, and PSSM-IC approaches by approximately 9%. At each specificity level, the true positive rate of our BoCaTFBS method is clearly higher than that with the other methods, whereas at each sensitivity level the false-positive rate of our BoCaTFBS method is lower than that of the other four methods. The consensus-IC approach still performs the worst, although it gains improvement by incorporating the per-position IC.

**Figure 4:**
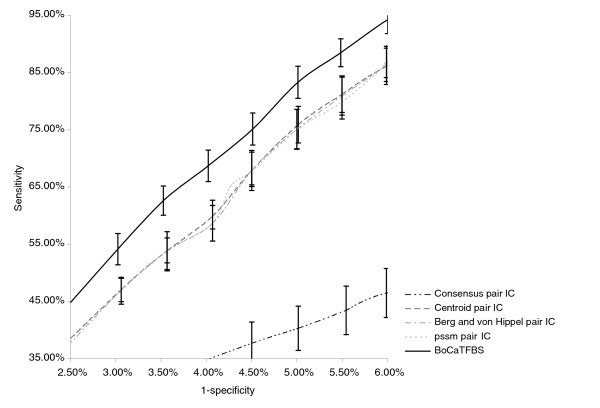
ROC curves comparing BoCaTFBS with centroid-pair IC, BvH-pair IC, PSSM-pair IC, and consensus-pair IC methods. The latter four methods are the four traditional methods incorporating both pair-wise correlation (full scope) and per-position information content (pair IC) [15]. The error bars are 95% confidence intervals. Our BoCaTFBS method noticeably outperforms the other four advanced methods. BvH, Berg and von Hippel; IC, information content; PSSM, positional specific scoring matrix; ROC, receiver operating characteristic.

The performance of our BoCaTFBS method and the other four methods incorporating both the local pair-wise correlations and per-position information content (pair IC) was evaluated in Figure 4. Although the centroid-pair IC, BvH-pair IC, and PSSM-pair IC gain some improvement over their simpler counterparts, our BoCaTFBS method still consistently has the best performance. For example, at the 5.5% false-positive rate, our boosted cascade method outperforms the centroid-pair IC, BvH-pair IC, and PSSM-pair IC approaches by about 7% to 8%.

### Demonstration of the value of non-binding information from ChIP-chip experiments

ChIP-chip experiments distinguish between binding regions and nonbinding regions for transcription factors [8]. Although the binding regions can only be narrowed down to thousands of nucleotides instead of precise sites, the nonbinding regions from these experiments provide useful information for identifying TFBSs.

**Figure 5:**
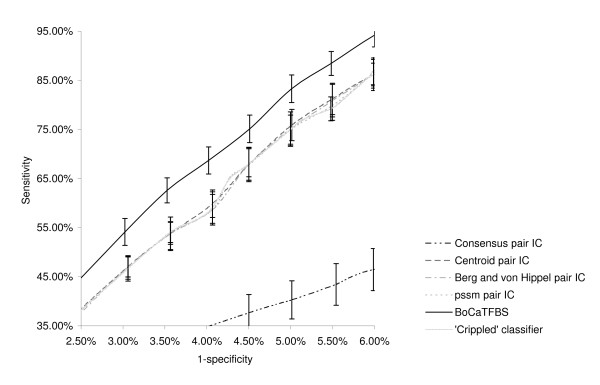
ROC curves showing the classification results for 'crippled' BoCaTFBS versus those of BoCaTFBS. In this comparison we used a 'crippled' classifier built without negative information from ChIP-chip data (dense discrete points in the graph), and compared the performance with that of our BoCaTFBS method using nonbinding site information from ChIP-chip experiments. The error bars are 95% confidence intervals. The results from traditional methods are also shown. ChIP, chromatin immunoprecipitation; ROC, receiver operating characteristic.

We evaluated the contribution of the negative information from ChIP-chip experiments to the prediction capability of a classifier. We did this by comparing the performance of the normal BoCaTFBS built with ChIP-chip data and a specially 'crippled' classifier built without the negative information from ChIP-chip data. For this 'crippled' classifier, we still used the 52 NF-κB (p65) binding sites [38] as the positive dataset. However, for the negative data pool for cascade training, we selected a total of 99,837 ten-nucleotide segments randomly from among 16,944,132 DNA segments tiled on chromosome 22 in the experimental design reported by Martone and coworkers [8]. That is, we picked negatives randomly from the segments used in the ChIP-chip experiment without knowing their actual binding results in the ChIP-chip experiment. The 52 known binding sites are excluded from this negative picking process. Both the positive dataset and negative data pool were utilized for 10-fold cross-validation and ROC curve calculation. As shown in Figure 5, at each specificity level the sensitivity of this 'crippled' BoCaTFBS prediction without correct negative samples from ChIP-chip experiments is about 7% to 8% below our normal BoCaTFBS prediction using nonbinding information from ChIP-chip experiments. Also, the results show that there is no improvement using our TFBS prediction method without nonbinding information from ChIP-chip experiments against other prior methods (centroid-pair IC, BvH-pair IC, and PSSM-pair IC). The results indicate that ChIP-chip experiments provide useful and discriminative information for our TFBS prediction method.

### Applications to the ENCODE project and further comparisons

In this section, we describe how we applied our BoCaTFBS method to the ENCODE regions of the human genome. These ENCODE regions were selected because they are intensively studied and we can investigate a variety of different transcription factors present in them. They provide an ideal platform for assessing the scalability and applicability of the method to the entire genome. The ongoing ENCODE project is making more human genome-wide ChIP-chip experimental data available [39]. Furthermore, we compared BoCaTFBS with other benchmarks, including the single boosting method, on the ENCODE regions.

Three transcription factors (*Sp1*, *cMyc*, and *P53*) datasets were retrieved from the work of Cawley and coworkers [44]. To obtain the positive training set, we used Clover, a program for identifying functional sites in DNA sequences [45], on the ChIP-chip binding regions (*P *< 10^-5^) to acquire the putative binding sites on these regions. The source of motifs is the JASPAR CORE collection of eukaryote TFBS patterns [46]. To avoid introducing more noise, we set a stringent threshold using a Clover *P *value of 0.01, which indicates the probability that the motif's presence in the target set can be explained just by chance, to retrieve these binding sites. The putative binding sites on chromosome 22 were retrieved by Clover in this way. There are 173 *Sp1 *binding sites, 627 *cMyc *binding sites, and 43 *P53 *binding sites identified in these regions on chromosome 22. Moreover, the nonbinding sites were retrieved based on the chromosome 22 sequence (14 September 2001, sequence 'release 3') [47], which is available from the Human Chromosome 22 Project website [48]. To simplify the problem, the preprocessing also included the application of RepeatMasker [49], a program that screens DNA sequences for interspersed repeats and low complexity DNA sequences [47]. There are a total of 34,344,351 *cMyc *nonbinding sites, 34,539,027 *Sp1 *nonbinding sites, and 34,566,391 *P53 *nonbinding sites on chromosome 22. For simplicity, a sliding window of five nucleotides was applied. Therefore, there are 6,869,066 *cMyc *nonbinding sites, 6,907,805 *Sp1 *nonbinding sites, and 6,913,291 *P53 *nonbinding sites. Both the binding sites and nonbinding sites were used for the training of the algorithms and cross-validation.

We compared our BoCaTFBS method with other methods on these three transcription factor datasets. The detection results of the binding sites on chromosome 22 for all of these three transcription factors (at false-positive rate 0.001) are shown in Table 1. The parameters were set empirically: the size of negative pool (δ) was set at 2000 arbitrarily; 25 cascade stages and 35 boosting steps for each stage were set for the *cMyc *BoCaTFBS learner; 20 cascade stages and 28 boosting steps for each stage were set for the *Sp1 *BoCaTFBS learner; and three cascade stages and 25 boosting steps for each stage were set for *P53 *BoCaTFBS learner. Moreover, because there was a memory insufficiency problem for a single boosting learner to train over all the negative data, we trained the single boosting learner from the positive training set and a fairly large (50,000) negative training subset. The number of iterations for the single boosting learner is the number of cascade stages multiplied by the number of the boosting steps per stage correspondingly. The results indicate that our BoCaTFBS method and the single boosting method performs consistently better than PSSM, centroid and BvH methods, and the improved variants reported by Singh and coworkers [15] (the consensus method performs consistently worse than all other methods as expected). The findings indicate that the discriminative methods (BoCaTFBS and single boosting method) take account of the discriminative features extracted from nonbinding sites, in addition to the information from binding sites.

Thus, our BoCaTFBS method and the single boosting method are capable of providing more accurate and delicate detection of the binding sites. Moreover, BoCaTFBS performs better in ENCODE applications than the single boosting method trained on 'reduced-to-fit' datasets. This indicates that an intelligent subsampling strategy embedded in BoCaTFBS cascade is more robust and efficient than a static 'reduce-to-fit' sampling. Boosting is known as a sequential procedure that is efficiently applicable only to relatively moderate datasets [41,42]. A straightforward sampling over a massive volume of data will possibly lose information and potentially become biased. BoCaTFBS intelligently re-samples and discards the 'easy negatives' rapidly through the cascade process (see Materials and methods, below). It avoids training over all the massive negative data in the repetitive learning process and is able to take more complete negative information into account through the cascade.

## Discussion

The position-specific scoring matrix technique is the basis for the majority of the TFBS prediction methods. However, this technique does not explicitly deal with negatives. Our BoCaTFBS method uses the nonbinding site information and improves the prediction accuracy of binding site identification. BoCaTFBS also incorporates the positional information and inter-dependence between positions. There is an abundance of nonbinding information available from ChIP-chip and other high-throughput experiments. BoCaTFBS provides an efficient and scalable method, and serves as a powerful complementary tool for experimental studies for identifying potential target genes of a given transcription factor. We foresee that a combination of computational searches and experiments will become an efficient approach for the identification of TFBSs.

We compared our method with a number of important preceding methods. In particular, we compared our method with four levels of benchmarks. First, we included in our comparison relatively simple traditional methods such as PSSM. We observed that our method achieves a clear improvement over these traditional methods. Second, we compared BoCaTFBS with enhanced versions of traditional methods that incorporate per-position IC and inter-positional relationship. We can see that these enhanced methods exhibit better performance than their simpler counterparts, but they proved less effective than our method. We next compared our method with the 'crippled' version of our classifier without negative information from ChIP-chip data. This resulted in inferior performance compared to the normal BoCaTFBS, which does incorporate the negative information. This outcome indicates that our method's improvement is contingent upon the negative information from the ChIP-chip assays. Finally, we applied our BoCaTFBS method to large-scale ENCODE data. In contrast to single boosting algorithms, which cannot scale to deal with massive datasets such as the human genome, the BoCaTFBS method's cascade structure adopts an intelligent data subsampling strategy to build an efficient TFBS identification framework that is scalable to the whole genome applications.

Our benchmark results indicate that our *BoCaTFBS *method outperforms the four traditional methods and their advanced variants in terms of sensitivity and specificity. Our method correctly identifies many transcription factor binding regions in human chromosome 22 based on the results of ChIP-chip experiments. Potentially, the optimized Markov chain method may be slightly more effective than the profile method (PSSM). Ellrott and coworkers [22], in fact, reported a 71% success rate on a small subset of their predictions in identifying the hepatocyte nuclear factor 4α binding site. However, we were unable to conduct a comparison of their technique with ours in detail because of the lack of accessibility of the optimized Markov chain code.

BoCaTFBS not only utilizes the massive amount of nonbinding information but also incorporates the positional information and interdependence information in creating a unified theme for TFBS prediction. It provides an integrative tool to search for TFBSs in the genome.

There are three major differences between our BoCaTFBS method and the MotifBooster approach proposed by Hong and coworkers [40]. First, MotifBooster constructs an 'ensemble' motif model that scores and classifies the bound and nonbound yeast ChIP-chip regions given a motif seed, whereas our BoCaTFBS method aims to classify the precise binding sites and massive nonbinding sites based on the human genome-wide ChIP-chip experiments. Second, the base classifier for MotifBooster is based on position-specific scoring matrix, whereas BoCaTFBS uses alternating decision trees (ADTBoost) within the cascade, which directly takes into account inter-position correlations as well as positional information. Finally, and most importantly, MotifBooster uses a standard boosting algorithm [42] that does not scale to massive datasets [42]. Our BoCaTFBS method adopts a boosted cascade framework [41], which provide an efficient and scalable method for massive and highly unbalanced datasets. Therefore, BoCaTFBS has wide application in genome-wide studies.

Currently, the ENCODE project is creating an increased availability of massive ChIP-chip datasets. More ChIP-chip 'tracks' will be available from the ENCODE browser for UCSC human genome assembly [50-52]. This trend has motivated us to develop fast, scalable, and accurate approaches to ChIP-chip data analysis and binding site recognition. The boosting technique has proved to be a good solution for differentiating true binding targets in ChIP-chip data from yeast [40], which has a small genome of only 16 megabases (Mb) of DNA. However, a single boosting classifier has limitations on massive datasets, because the size of the dataset can be a bottleneck. One has to load sequentially and train on all of the 'massive training samples' repetitively during each step in trying to learn a single complex classifier [42]. This is impractical in many situations in human genomic research. Even in our simplified example, where we only focused on ChIP-chip experimental results of the second smallest human chromosome (chromosome 22), the enumeration of negative segments from NF-κB nonbinding regions already takes 809 Mb in FASTA format [8]. Furthermore, the Human Genome Project has finished about 3 gigabases of sequence (released April 2003). Finally, the highly skewed distribution of training samples makes the classifier biased toward the dominant class, which is undesirable. The expanding large-scale human genomic ChIP-chip datasets present a challenge that demands scalable and efficient methods.

To handle massive datasets, it is necessary to bypass the need for loading and repetitively training over the entire dataset in the memory of a single computer as standard boosting requires. Notably, the boosted cascade employed in our BoCaTFBS method is computationally efficient by training only over small subsets and cascading its training and evaluation. In particular, the technique of boosted cascade has proved to perform extremely quickly in domains where the distribution of the positive and negative examples is highly skewed [41,53]. The key idea of the boosted cascade is that smaller and therefore more efficient boosted classifiers based on a small subset instead of the whole dataset can be constructed to reject many of the negatives while detecting most of the positive instances. In the training, simple classifiers are utilized to exclude the majority of the negatives and focus on only false positives before more complex classifiers are called upon to achieve a low false-positive rate. Therefore, BoCaTFBS avoids storing and training over all the massive amount of negative information in the repetitive boosting process and achieves optimal efficiency. In the testing, the cascade also attempts to reject as many negatives as possible in the earliest stages. Thus, the boosted cascade is one of the most efficient algorithms when the distribution of the positive and negative examples is highly unbalanced, like the TFBS identification problem. The computational efficiency and scalability of our BoCaTFBS method is very important given the large sizes of chromosomes in the genome that need to be scanned. As the running time of our *BoCaTFBS *method is in minutes when applied to our experiments on chromosome 22, we can estimate that our method will most likely finish in hours when applied to the whole genome.

## Conclusion

In order to understand the molecular mechanisms of gene regulation, a robust method is required to discriminate TFBSs from nonbinding sites on a genomic scale. Experimental methods such as ChIP-chip experiments, although gaining great success, remain time-consuming, expensive, and noisy. Traditional computational methods for binding site identification, such as consensus sequences, profile methods, and hidden Markov models, are known to generate high false-positive rates when applied on a genome-wide basis. They are based on training only with positive data, which are small number of known binding sites. Thus, we were motivated to propose a new computational method (BoCaTFBS) to discover TFBSs that combines the noisy data from ChIP-chip experiments with known positive binding site patterns.

Our method uses a boosted cascade of classifiers, in which each component is an individual alternating decision tree (an ADTBoost classifier). It uses known motifs, taking advantage of the inter-positional correlations within the motifs, and it explicitly integrates the massive amount of negative data from ChIP-chip experiments. We tune BoCaTFBS to reduce the false-positive rate when applied genome-wide and use the cascade for optimum computational efficiency, an important consideration for genome-scale applications. We show that *BoCaTFBS *outperforms many traditional binding site identification methods (such as profiles) in terms of sensitivity and specificity. We also show how its improvement is directly tied to the inclusion of the negative information from ChIP-chip experiments. Moreover, we show that BoCaTFBS can be successfully applied in the ongoing ENCODE project, which aims to identify all functional elements in the human genome sequence.

Given the scale of the human genome and the noisiness and error in both the purely computational predictions and the ChIP-chip experiments, we feel that this hybrid approach, which combines ChIP-chip data with efficient computational learning, provides promise for the future. We envision that when more data are available from larger experiments, we will be able to refine our classifier further, thereby achieving a lower false-positive rate.

## Materials and methods

In order to predict the TFBSs, we employ a form of supervised machine learning: a number of ADTboost learners coupled in a boosted cascade. ADTboost is a special extension of AdaBoost. For clarity, we introduce our algorithm for identifying TFBSs in a logical order of AdaBoost, ADTboost, and Boosted Cascade (see the supplementary website [54]).

Initially, we start with the following type of data. In the training stage of TFBS prediction, its input is (x_1_, y_1_) ... (x_n_, y_n_), where each x_i _belongs to an instance space of a string of four nucleotides (A, T, G, C) corresponding to each position in the TFBSs and y_i _belongs to label set Y = {+1,-1} (where +1 represents 'binding' and -1 represents 'nonbinding' for a given transcription factor).

### AdaBoost

**Figure 6:**
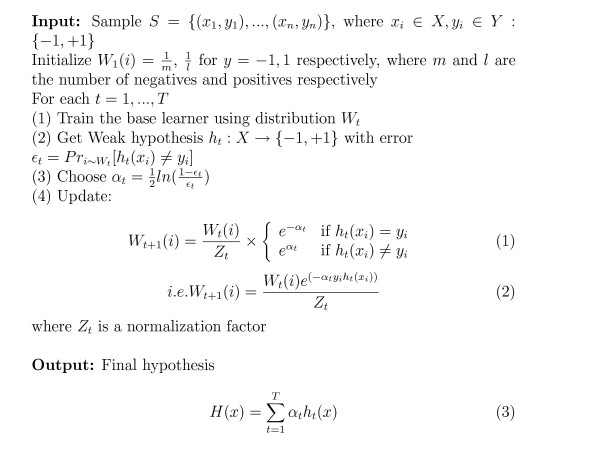
The boosting algorithm: AdaBoost.

In general, boosting is a method for improving the accuracy of any given learning algorithm. AdaBoost solved many practical difficulties of earlier boosting methods [55,56]. It takes the training data as described above. AdaBoost repeatedly calls a given base learning algorithm in t rounds. W_t_(i) represents the weight of the distribution on training example i on round t (set of weights over the training examples). At each iteration t, the base learner is utilized to find a weak hypothesis h_t_: X → {-1,+1} appropriate for the distribution. The weights will be updated. Usually, the weights of incorrectly classified examples are increased so that the base learner is forced to concentrate on the hard examples in the training set. The base learner is called again with new weights over the training examples, and the process repeats. At last, all the weak hypotheses are combined into a single, strong hypothesis using a weighted majority vote (see algorithm details in Figure 6).

### ADTboost

**Figure 7:**
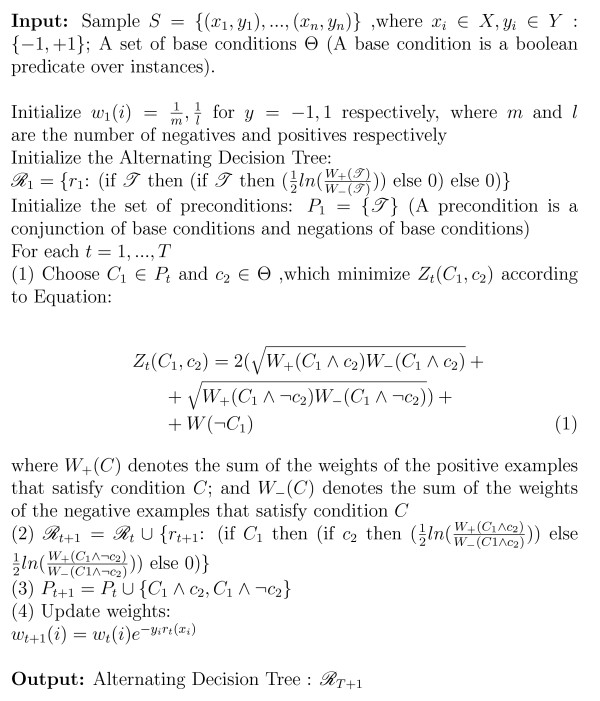
ADTboost algorithm.

In this report we utilize a special extension of AdaBoost called the alternating decision tree (ADTboost). AdaBoost is used to learn the decision rules constituting the tree and to combine these rules through weighted voting [57] (refer to algorithm details in Figure 7). The resulting tree is in a well presented, intuitive format. It generates decision rules that are easily interpretable.

**Figure 8:**
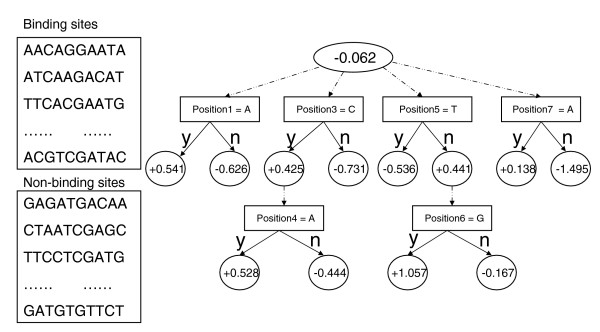
A simple example of the alternating decision tree (ADTboost). The alternating decision tree contains splitter nodes (squares, associated with a test) and prediction nodes (circles, associated with a value). Each prediction node represents the results of a weak prediction rule. The number in the prediction nodes (circles) defines the contributions to the prediction score. In this example, negative contributions are evidence of nonbinding, whereas positive contributions are evidence of binding. The position and nucleotide features are used for constructing the weak prediction rule. In order to evaluate the prediction for a particular DNA sequence, we begin from the top node and follow the arrows down. We sum all the values at all the prediction nodes reached. This sum represents the prediction score, and its sign is the prediction by default. For instance, in the DNA sequence of AACGCTAATA, the nucleotide at position 1 is A, the nucleotide at position 3 is C, position 4 is not A, position 5 is not T, position 6 is not G, and position 7 is A. Applying the alternating decision tree in the figure to this, we derive the following prediction nodes: +0.541 (from A at position 1), +0.425 + -0.444 (from C at position 3 followed by not A), +0.441 + -0.167 (from not T at position 5, followed by not G), and +0.138 (from A at position 7). Notice that we do not refer to position 2 and other positions because they are not relevant to the rules here. The overall sum of all the nodes is +0.803, a confident score indicating that this is predicted to be a binding site.

AdaBoost generates the 'alternating decision tree' from the training data (as described above) as the detailed algorithm in Figure 7 shows [57]. In the testing stage, the alternating tree maps each instance to a real valued prediction, which is the sum of the predictions of the base rules in its set along the related paths in the tree that actually incorporates positional information and inter-positional relationships by logical combination. The classification of an instance is the sign of the prediction. In order to explain the concept in a simple way, we utilize a straightforward example. This example, shown in Figure 8, predicts some molecules' DNA-binding sites based on the sequence after training on both binding and nonbinding sites.

### Boosted cascade

For TFBSs, there are a number of binding sites (positive training sets) and a significantly large quantity of nonbinding sites (negative training sets). In machine learning, besides the scalability and efficiency issues, an imbalance problem also arises when there is a great size disparity between the positive and negative training sets. This imbalance problem could lead to accurate prediction of the over-represented class but unfortunately incorrect prediction of the under-represented class. To solve these problems, an algorithm for constructing a cascade of classifiers [41] was developed, achieving excellent detection performance while radically reducing computation time. Our BoCaTFBS method uses such a cascade of classifiers for the TFBS identification problem.

To train a cascade of classifiers, we can construct boosted classifiers that reject many of the negative instances while detecting almost all the positive instances; specifically, the threshold of a boosted classifier can be adjusted so that the false-negative rate is close to zero. The process of training a cascade of classifiers is an iterative process. First, randomly choose a negative training subset δ from the negative training set *N*. Second, train a classifier C(i) with a positive training set P and the chosen negative training subset δ using ADTboost. Third, adjust the threshold to minimize the false negatives (to make all the positive training samples predict 'positive'). Fourth, screen the negative training set *N *by the adjusted threshold in step three to arrive at set *N'*. Fifth, redefine *N' *to be the negative training set *N*. Finally, repeat this process above until some predefined criteria is met to stop the cascade.

**Figure 9:**
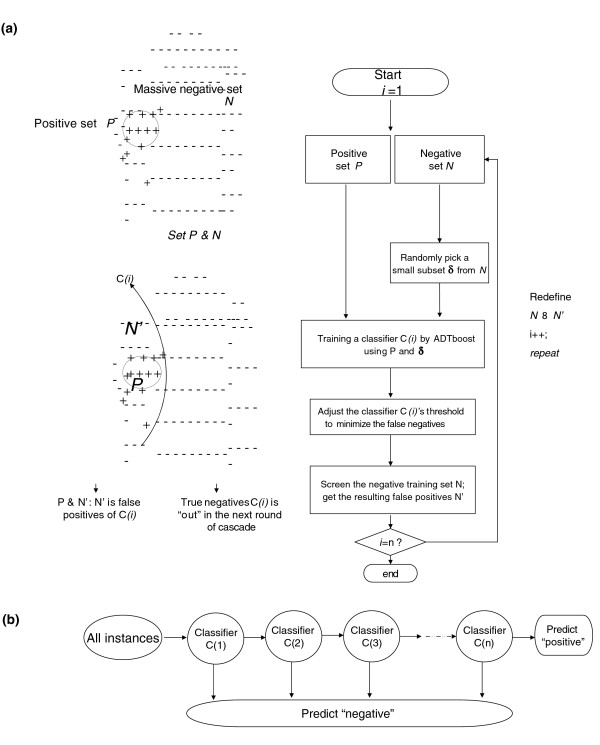
Boosted cascade. **(a) **Flowchart of training a cascade of classifiers. **(b) **Detection cascade. A series of classifiers are applied to each instance. Each layer of classifier eliminates negative examples. This cascade of classifiers achieves increased detection performance while radically reducing computation time.

The detection process is also represented as a 'cascade'. A positive result from the first classifier triggers the evaluation of the second classifier, which has also been adjusted to achieve very high detection rates. A positive result from the second classifier triggers a third classifier, and so on. A negative outcome at any point leads to immediate rejection. Figure 9 panels a and b depict the training and detection cascades, respectively.

### Datasets for application of the method

We utilized transcription factor NF-κB (p65) as the test case for our methods. The 52 NF-κB (p65) binding sites [38] are used as the positive dataset. For simplicity, a total of 99,837 nonbinding sites for NF-κB from ChIP-chip experimental data on human chromosome 22 [8] were utilized as the negative data pool for cascade training. These 99,837 nonbinding sites were randomly chosen from the 16,775,258 nonbinding sites from the ChIP-chip experiments to facilitate computation. In the training cascade, each negative subset δ, consisting of 3000 sequences, was randomly chosen from the screened negative training set *N*. Both the positive dataset and negative data pool were utilized for repeated tenfold cross-validation and ROC curve analysis. The ROC curves for every prediction method were averaged vertically based on repeated tenfold cross-validation in order to evaluate the performance of each prediction method [58].

We also applied our method to the ChIP-chip datasets from the ENCODE project. Three transcription factors (*Sp1*, *cMyc*, and *P53*) datasets were retrieved from the work of Cawley and coworkers [44]. The ChIP-chip binding regions of these three new transcription factors are available on the world wide web [59].

### Other TFBS prediction methods compared

We compared the performance of our method with those of other TFBS prediction methods and their advanced extensions (Table 2). We only very briefly describe these methods as follows. At first, we considered four basic methods (consensus, PSSM, BvH, and centroid), and then considered enhancements to those methods and a crippled version of BoCaTFBS, and finally MotifBooster.

#### Consensus

The consensus method is the simplest method for TFBS prediction. For each position, if the frequency of the most frequent base is larger than 0.5, then this base is the consensus base for the position. Otherwise, if the sum of the frequencies of the most frequent base and the second most frequent base is larger than 0.75, then these two bases are the consensus bases. If neither of the preceding is true, then there is no consensus sequence base for the position. The score of the predicted sequence is obtained by counting the number of times the base of the sequence agrees with the corresponding consensus base for each position [60].

#### Profile (or PSSM)

This method assumes independence between positions and computes the log-odds score for a potential binding site. Bayesian estimate was utilized to estimate the zero frequency case [61].

#### Berg and von Hippel (BvH)

This method is a statistical mechanics based method that makes the connection between base-pair statistics of a set of sites and its binding free energy [62,63].

#### Centroid

This method scores a sequence by computing the average shared identity between this sequence and every known binding site sequence for a given transcription factor [15].

#### Information content and local pair-wise correlation

Singh and coworkers [15] introduced information content (IC) and local pair-wise correlation concept into these four basic methods to augment the prediction performance. They extended the four methods above by incorporating pair-wise dependencies. The notion of scope delimits the pairs that are considered correlated into the scoring scheme. IC is an important concept based on the information theoretic notion of entropy. The entropy of a binding site position expresses the number of bits necessary to describe that position, and the information content of a position is defined as the difference between the position's maximum possible entropy and its entropy. They incorporated the per-position information content as a multiplicative factor to weigh the contribution of each position (or pair of positions) in scoring a target binding site sequence. Based on the benchmark results on *E. coli *TFBS predictions reported by Singh and coworkers [15], the use of per-position IC improves the performance of the four traditional methods in many cases, and the best prediction results were obtained by incorporating both IC and local pair-wise correlations.

#### 'Crippled' BoCaTFBS

To evaluate the contribution of negative information from ChIP-chip experiments, we also use a 'crippled' BoCaTFBS as a benchmark. The only difference between 'crippled' and normal BoCaTFBS is that the 'crippled' one is built without the negative information from ChIP-chip data.

#### MotifBooster and related method

Hong and coworkers [40] introduced a standard boosting approach to model transcription factor-DNA binding using yeast ChIP-chip data. It constructs an ensemble motif model, which scores and classifies bound and nonbound regions in ChIP-chip experiments. The base classifiers of MotifBooster are derived from PSSMs. BoCaTFBS, in contrast, classifies binding sites instead of binding regions. To compare BoCaTFBS with single boosting method, we used ADTboost, a variant of single boosting algorithm, as a benchmark. ADTboost takes into account not only positional preferences but also inter-positional relationships directly as features in classifying binding versus nonbinding sites.

## Additional data files

The following additional data are available with the online version of this paper. Additional data file 1 shows a BoCaTFBS classifier trained over NF-κB ChIP-chip experimental data (the complete version of that shown in Figure 1). Additional data file 2 illustrates the relationship between BoCaTFBS with a single boosting classifier on a moderate dataset. Additional data file 3 lists 627 *cMyc *binding sites, 173 *Sp1 *binding sites, and 43 *P53 *binding sites on chromosome 22. The supplementary website is available at [54].

## Supplementary Material

Additional data file 1 A BoCaTFBS classifier trained over NF-κB ChIP-chip experimental data (the complete version of that shown in Figure 1). Click here for file

Additional data file 2 Relationship between BoCaTFBS with a single boosting classifier on a moderate dataset. Click here for file

Additional data file 3 List of 627 *cMyc *binding sites, 173 *Sp1 *binding sites, and 43 *P53 *binding sites on chromosome 22. Click here for file

## Figures and Tables

**Table 1:** BoCaTFBS application in ENCODE projects

Transcription factor	Methods	TFBSs detected correctly		
		
		Original	IC	Pair IC
*cMyc*	PSSM	234	234	261
	Centroid	232	236	241
	Berg and von Hippel	245	247	252
	Consensus	154	219	221
	Single boosting	347		
	BoCaTFBS	444		

*Sp1*	PSSM	86	86	90
	Centroid	93	93	104
	Berg and von Hippel	107	109	115
	Consensus	62	68	68
	Single boosting	119		
	BoCaTFBS	123		

*P53*	PSSM	16	17	29
	Centroid	15	15	23
	Berg and von Hippel	16	19	29
	Consensus	7	12	17
	Single boosting	30		
	BoCaTFBS	35		

IC, information content; PSSM, positional specific scoring matrix; TFBS, transcription factor binding site.

**Table 2:** Traditional TFBS prediction methods and their extensions

Method	Incorporate per-position IC	Incorporate both per-position IC and pair-wise correlation
Consensus	Consensus-IC	Consensus-pair IC
Centroid	Centroid-IC	Centroid-pair IC
Berg and von Hippel	Berg and von Hippel-IC	Berg and von Hippel-pair IC
PSSM	PSSM-IC	PSSM-pair IC

The details of the scoring schemes are described in the report by Signh and coworkers [15]. In our calculation, we use full scope for incorporating pair-wise correlation to the four TFBS prediction methods. IC, information content; PSSM, positional specific scoring matrix; TFBS, transcription factor binding site.
