# Supplementary material for: BoCaTFBS: a boosted cascade learner to refine the binding sites suggested by ChIP-chip experiments
Source: Genome Biol. 2006 Nov 1;7(11):R102. doi: 10.1186/gb-2006-7-11-r102 (PMC1794589; doi:10.1186/gb-2006-7-11-r102)
Supplement: Additional data file 1 — A BoCaTFBS classifier trained over NF-κB ChIP-chip experimental data (the complete version of that shown in Figure 1). [file gb-2006-7-11-r102-S1.pdf]

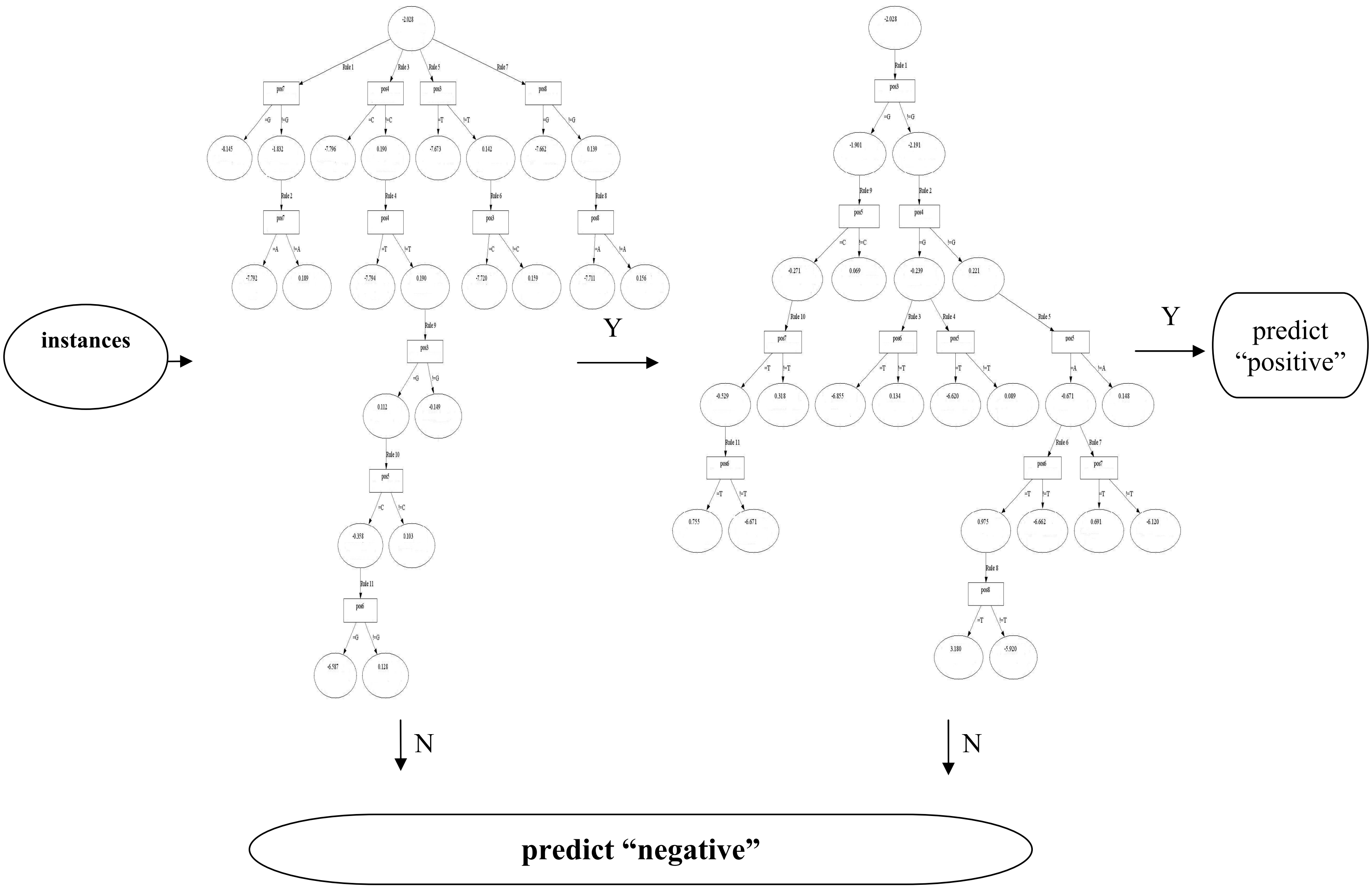
instances
Y
Y
predict "positive"
N
N
predict "negative"

Figure: A *BoCaTFBS* classifier trained over NF-κB ChIP-chip experiments data.

Two cascade stages and 12 features in each stage were empirically predefined for NF-κB binding sites prediction. This cascade predictor was tested by cross validation, and shown 82% sensitivity (true positive rate) at the 5% false positive rate level. It seems that the *BoCaTFBS* classifier is built on discriminative features (which differentiates positives from the chosen negative training set), by differentiating the binding sites from the non-binding sites. For example, in stage 1, the sequence in which position 4 is not C is more likely to have more binding propensity. The consensus sequence of binding sites is GGGRNNYYCC (R is purine, Y is pyrimidine, and N is any nucleotide), which does not show strong propensity for “not C” at position 4. In contrast, the single ADTboost classifier at the first cascade stage shows 71% true positive rate at 5% false positive rate level. It seems that the further stage refines the positives predict and increases the true positive rate over the prior cascade stage. We need to clarify that a technical obstacle for the classifier cascade is that for each classifier: (1) it is built upon a random small subset of the over-represented class at each stage, and (2) each classifier is dependent on the results of the classifiers in the previous stages. Thus, the classifier cascade represented is not meant to stabilize "in appearance", and the exact discriminative features to chosen at each step are not stable due to the random nature of the cascade, particularly in the random subset selection process of the negative samples for training.
