# Supplementary material for: BoCaTFBS: a boosted cascade learner to refine the binding sites suggested by ChIP-chip experiments
Source: Genome Biol. 2006 Nov 1;7(11):R102. doi: 10.1186/gb-2006-7-11-r102 (PMC1794589; doi:10.1186/gb-2006-7-11-r102)
Supplement: Additional data file 2 — Relationship between BoCaTFBS with a single boosting classifier on a moderate dataset. [file gb-2006-7-11-r102-S2.pdf]

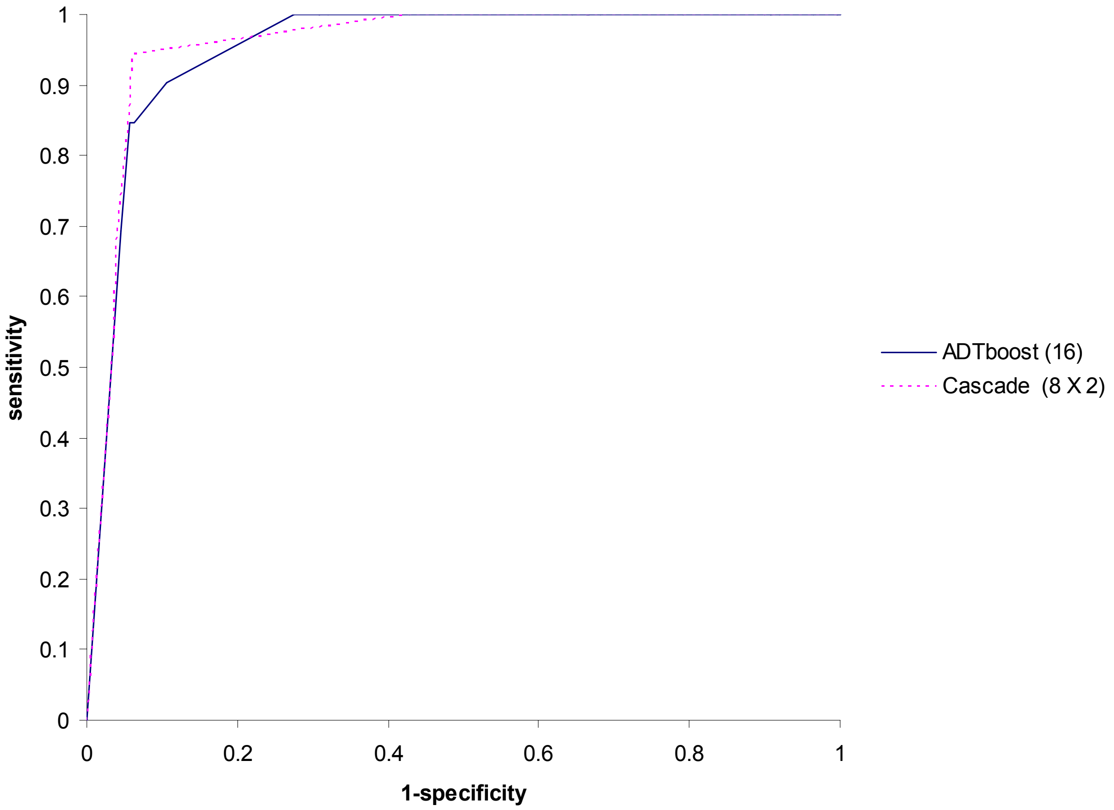

"To Cascade or Not? This is a Problem"

Figure: Relationship between *BoCaTFBS and a single ADTboost Classifier on a moderate dataset*

(The cascade here is a two-stage classifiers with eight features each stage, while ADTboost is with sixteen features).

We empirically compared the performance between a single ADTboost and *BoCaTFBS* classifier. The cross-validation of these two classifiers was performed on the same small dataset consisting of 52 TF binding sites and 3326 non-binding sites. The results show that cascaded classifier has close performance with a single ADTboost classifier. It shows that boosted cascade maintains the excellent discriminative capability of the classical boosting algorithms.

However, there are massive non-binding sites in human genome, which challenges the single boosting classifier with both scalability and efficiency issues [41, 42]. This is because boosting is an algorithm that has to sequentially load and train on all the "massive training samples" repetitively during each step and try to learn a single complex classifier. A straightforward static sampling over such large datasets will potentially lose information and result in biased classifier, as shown in our manuscript. The cascade structure, which we adopted in our *BoCaTFBS*, provides a practical solution to this situation. It obviates the need for massive data loading and training, focuses on the harder samples and promising

regions rapidly (please refer to *Methods*), while still maintaining the excellent discriminative ability of general boosting algorithms.
