# Supplementary material for: BoCaTFBS: a boosted cascade learner to refine the binding sites suggested by ChIP-chip experiments
Source: Genome Biol. 2006 Nov 1;7(11):R102. doi: 10.1186/gb-2006-7-11-r102 (PMC1794589; doi:10.1186/gb-2006-7-11-r102)
Supplement: Additional data file 3 — List of 627 cMyc binding sites, 173 Sp1 binding sites, and 43 P53 binding sites on chromosome 22. [file gb-2006-7-11-r102-S3.pdf]

TRANSCRIPTION FACTOR	BINDING SITES SEQUENCES
cMyc	atcacatgga<br>accatgtagt<br>acaccgtggc<br>gccaggtggt<br>ggcctgtggt<br>agcacacgga<br>aacacatgtt<br>tccaggtggt<br>gcaatgtggt<br>cgcacgtgca<br>ataacatggt<br>atcacgaggt<br>ttcacgtgga<br>tgcacgtgga<br>agcccgtgcc<br>tacacgtgcc<br>ggaatgtgga<br>agaaggtgga<br>gccacttggt<br>accacatagt<br>agaacgtgac<br>agcacgtggc<br>agcacgttct<br>cccacggggt<br>aacacgggtt<br>tccacgtgaa<br>gccacgtgca<br>tccacgcggt<br>ggaatgtggt<br>gccatgtggg<br>accatttgat<br>ggtacgtggc<br>accacgccgt<br>agcaggtggt<br>cgcacgtggg<br>cccatgtggt<br>gccaggtgtt<br>accaagtgcc<br>gccacgaggc<br>tgcccgtggt<br>gtcacgtgac<br>gtcacgtgac<br>tccacatggc<br>agaaggtggc<br>accacgggca<br>gcctcgtggc<br>gccatctggt<br>ggcacttggt<br>aacacctggc<br>gccacgtgcc<br>cccacatggc<br>accaagtggc<br>aagacgtggc<br>accacacggc<br>gccatgtgcc

	aacacgtgcc<br>gccacgtgtt<br>ggcacttggc<br>atcacgggct<br>agaacatgga<br>aacatgtgtt<br>ggcacatggc<br>tgcacgtgga<br>cccacgtgct<br>cccacgtgac<br>gccaggtgtt<br>accaagtgcc<br>gccacgaggc<br>tgcccgtggt<br>gccatgtgga<br>gtcacgtgac<br>gtcacatggc<br>tccacatgga<br>gggacgtggc<br>acctcgtgat<br>aggaggtggt<br>accacctggt<br>accacctggt<br>gccacgtgcc<br>cacacgcggt<br>agcaggtgga<br>ggcacgtggc<br>gcaacatggt<br>accacgtggt<br>atcacatggc<br>aacacgtgaa<br>gccacatgtt<br>agcacgtggt<br>tgcaggtggc<br>gccacgtgtt<br>gacacctggt<br>accacgtgga<br>gccatgtgct<br>accaggtggc<br>cccacttggt<br>accacgtggg<br>acctcgtgat<br>accaggtgac<br>tgcatgtggc<br>agcatgtgca<br>acctcgtgat<br>accacattgc<br>ggcatgtggt<br>cccatgtgga<br>agcagggggt<br>gtgacgtggt<br>agcacttgtc<br>tccacctggc<br>gccatgtgct<br>accatgcggc<br>gccacacggt

	gccacgtggc<br>tccacatggc<br>agaaggtggc<br>accacgggca<br>gcctcgtggc<br>gccatctggt<br>accacatggg<br>gggacgtgga<br>agcgcgtggc<br>tccacttggt<br>ggcacttggt<br>accaagtgaa<br>agcagatggc<br>gccacatgca<br>gtcacgtgtt<br>aggacgtggt<br>accaggtggc<br>aacaggtgga<br>agcacgtggc<br>agaacatgga<br>aacacatggg<br>ggcaggtggt<br>gtcacgtggg<br>agcacgtggg<br>aggacgtgtc<br>ggaacgtgac<br>aacacgtgtt<br>gccacgtgct<br>gtcatgtgga<br>agcccgtgat<br>accaggtgga<br>accatatgga<br>gtcacgtgac<br>agaaggtggc<br>accacgggca<br>gcctcgtggc<br>gccatctggt<br>ggcacttggt<br>aacacctggc<br>gccacgtgcc<br>cccacatggc<br>ggcacgtgcc<br>accaagtggc<br>aagacgtggc<br>accacacggc<br>agcatgcggc<br>accacaaggc<br>tgcacgtggc<br>gccatgtgcc<br>gccacgtgtt<br>ggcacttggc<br>atcacgggct<br>agaacatgga<br>aacatgtgtt<br>ggcacatggc<br>tgcacgtgga

	cccacgtgct<br>cccacgtgac<br>gccaggtgtt<br>tacacgtgtt<br>accaagtgcc<br>gccacgaggc<br>gccatgtgga<br>ggcaggtggc<br>ggcatgtgcc<br>ggcacgtggc<br>atcacgaggt<br>gccatgtggt<br>accacatggc<br>acctcgtgat<br>gccacgtgcc<br>aacacctggc<br>agcacgtcct<br>agcacgtgca<br>tgcacgtgct<br>accacgagat<br>aaaacgtgcc<br>acctcgtgat<br>ggcacatggc<br>accacctggc<br>gccacatgat<br>cacacgtgct<br>aacacatgga<br>gtcacgtgca<br>aatacgtgta<br>gacacctggt<br>ggcacggggc<br>tccacatggt<br>ggaatgtggc<br>gccaggtgga<br>cccatgtggc<br>agcacgtggc<br>agcacgtggc<br>agcacgtggc<br>gacacctggt<br>ggaacgcgga<br>accacgggcc<br>accatgtgtc<br>agcacctgat<br>accacgggga<br>accacctgat<br>accacgggga<br>accacttgat<br>ggcaggtggt<br>accatgtggt<br>ggaacatggc<br>tgcacgtgca<br>accacatggt<br>gtcacgtggg<br>agcacgtggg<br>aggacgtgtc<br>gacaggtggc

	ggaacgtgac<br>aacacatgtt<br>gccacgtgct<br>tgcacttggt<br>gccacctggt<br>ggcacgtggc<br>aggatgtggt<br>ggcacgtgga<br>accaggtgtc<br>gtcatgtggc<br>accaggtggc<br>ggcacttgga<br>agcacatggg<br>cccacgtgat<br>atcacgaggt<br>ggcacgtgcc<br>tccacgtggt<br>atcacgtaga<br>gcaacgtgcc<br>agcaggtggt<br>ggcacatgtt<br>agcaggtggg<br>agcaagtgtt<br>accacgggac<br>gtcacgtggg<br>agcacgtggg<br>aggacgtgtc<br>gacaggtggc<br>ggaacgtgac<br>aacacatgtt<br>gccacgtgct<br>agcaaatggc<br>ggcacacggc<br>agcatgtgtc<br>gccaggtgga<br>agcaggtgtc<br>ggcacggggc<br>agcactttgt<br>ctcacgtggc<br>cccacgtgtt<br>gccacatggc<br>gacaggtggc<br>agcactttgt<br>ctcacgtggc<br>cccacgtgtt<br>gccacatggc<br>gccaggtgtt<br>gccacgaggc<br>accacgggtc<br>cacacgaggt<br>accaagtggt<br>ggcttgtggt<br>gggacatggt<br>cccacatggt<br>ggcatgtgcc<br>agaacatgga

	agaacgtgaa<br>agcacatggg<br>agcacgtgcg<br>accacatggg<br>accacagggt<br>agcccgtgaa<br>accacgtggg<br>cacacgtgac<br>aagacgtggt<br>agcaaggggt<br>cacacgtgac<br>agcacatgca<br>agaacttggt<br>cccacgtggc<br>agcacgtggc<br>agaacatgga<br>aaaatgtggt<br>aacatgtgtt<br>ggcacatggc<br>tgcacatgga<br>cccacgtgct<br>cccacgtgac<br>gagacgtggt<br>ccaacatggt<br>ggcacgtgca<br>tccacgtggt<br>atcacgtctt<br>accgcgtggc<br>agaatgtggt<br>cgcacgtgct<br>gagacgtggt<br>gccacttggt<br>ggcaggtgtt<br>tgcacttggt<br>gccacctggt<br>ggcacgtggc<br>aggatgtggt<br>gtcacgtggg<br>agcacgtggg<br>agcacatgcc<br>aggacgtgtc<br>gacaggtggc<br>ggaacgtgac<br>aacacatgtt<br>gccacgtgct<br>cccacatgga<br>atcatgtgga<br>aaaacgtggt<br>aacacgtgac<br>cccacatggt<br>atcacgaggt<br>ccaacatggt<br>accacatgaa<br>caaacgtggt<br>gccacttggc<br>accacatggc

	aacatgtgta<br>atcatgtgga<br>tccatgtggc<br>acctcgtgat<br>aacacgtgac<br>atcatgtgga<br>agcatgtggt<br>ccctcgtggt<br>accatgtgcc<br>accaggtgtc<br>ggcacgtgaa<br>cacacgtggc<br>accatctggt<br>gccacatgat<br>aacacgtgct<br>accacgtggc<br>accacttgga<br>tccacctggc<br>accacatggt<br>accacgtagc<br>gccatgtgtt<br>accacttagt<br>gtcacatggt<br>agcacatggc<br>gacacttggc<br>agcatgtgcc<br>ggcatgtggt<br>acaacgtggg<br>atcacttggc<br>agcacgtgtt<br>agaacgtgct<br>gacacctggt<br>acaatgtggc<br>ggcacgtggg<br>accacgagcc<br>acaacgtgct<br>gacacatggt<br>cccacgaggc<br>gccacgagtc<br>agcacgtgct<br>gccacctggg<br>gccacgttgc<br>agcacagggt<br>agcacctggc<br>agcacgtgcc<br>tgcacgtggc<br>accaaatggc<br>agcacctggc<br>gccacatgct<br>agcacatggt<br>tccatgtggt<br>agcatgtgtt<br>accaagtggc<br>gccacatggc<br>gtcacatggc<br>accatgtaga

	atcacgaggt<br>aacaggtggc<br>agcacatggc<br>accacctggt<br>ggcacatgga<br>agaacgaggt<br>ggcatgtgac<br>gccacgtggc<br>gccacgtggt<br>cgcacgtggg<br>cgcatgtggc<br>ggcacatgga<br>agcacctgac<br>gtcacatggc<br>tacacgtggt<br>ggcacttgga<br>accacctgga<br>ggcacgtgcc<br>agcatggggt<br>accacatgct<br>gccatgtggc<br>gccctgtggt<br>accaggtggc<br>atcacgtggt<br>atcacgtggt<br>aaaacgtgac<br>gacacgtggg<br>ggcacttggc<br>caaacgtgga<br>aacatgtggc<br>gacacgtgga<br>gccacgttgc<br>accacacgga<br>agcacctgga<br>agcaggtgtc<br>ccaacatggt<br>accacgtagt<br>aacaggtggt<br>tcctcgtggt<br>gacaagtggc<br>atcacatggg<br>accccatggc<br>agagcgtggc<br>agcatgtgtt<br>accacctggg<br>tgcaggtggt<br>agctcatggc<br>acccccttggt<br>agaacatgga<br>cgaatgtggt<br>agcacgagtt<br>acaatgtgtt<br>ggcacctgga<br>gtcacatggc<br>agcactttgt<br>agaacgcggt

	gtcacgtgac<br>cccacgtgct<br>tacatgtgga<br>gccacgtcgt<br>agcccgtgga<br>agcacctgct<br>accacctggt<br>tccacgtgca<br>gccacgaggc<br>acaatgtggt<br>aacacgttgc<br>gccacgtgca<br>gcaacgtgga<br>ggcacgtgcc<br>ggcacgtgcc<br>aacatgtgtt<br>agaacatggc<br>accactcggt<br>tcctcgtggt<br>gccatgtgac<br>cccacgtgcc<br>gacacttgga<br>ggcacggggc<br>ggcacatggt<br>tacatgtggc<br>ggcacttggt<br>ccaacatggt<br>accacgggtt<br>gccacgtgat<br>cacacgtgct<br>ggcaagtggc<br>aacacatggc<br>accacgaggc<br>atcaggcggt<br>accacagggt<br>accacagggc<br>agcacttgct<br>agaacgtggc<br>tccacgtggt<br>agtacatggc<br>acaacgtgta<br>cgcacgtgtc<br>gtcacgtgct<br>gccccgtgga<br>ccaacatggt<br>gtcacgtggt<br>gacgcgtggt<br>agcacctggc<br>gccacatgtt<br>cgcaggtggc<br>ggcaggtggt<br>aggacgtcgt<br>gccacgttga<br>aacaaatggt<br>gacacctggc<br>cacacgtggg

	gccaggtgga<br>atcacgtgct<br>cgcaggtggc<br>cccacgcggc<br>accatgtgat<br>agaacgtgcc<br>atcatgtggt<br>aacacatgct<br>cccacatgat<br>accaccaggt<br>tgcaggtgga<br>gacacctggc<br>ccaacatggt<br>accacgctgt<br>ggtacgtgct<br>acctcgtgat<br>ggcatgtgcc<br>atcacgaggt<br>agcatgtgac<br>ggcacgtgtg<br>agcacgtctc<br>cacacgtgga<br>ggcaggtgga<br>gccccgtggc<br>gaaacgtggc<br>agcaagtggt<br>cgcacgtggt<br>cccacgtggg<br>aactcgtggt<br>agcacttggc<br>atcacatgct<br>tccacctggc<br>ggcacatgga<br>agcacgtgtc<br>agtccgtggt<br>ggcatgtgga<br>gacacgtgca<br>gacacttggt<br>gccaggtgct<br>gacacgtgca<br>gacacttggt<br>atcaagtgga<br>accacgtgcg<br>ggcaggtggc<br>accaggtgat<br>tccatgtggc<br>gcaacgtgac<br>agcaggtgcc<br>accaggtggg<br>ggcacgttgc<br>ggcacatgga<br>agcaggtggc<br>ggaacgtgcc<br>ataatgtggt<br>agcacctgct<br>gccacatgct

	gccacatgac
	accatgtgtc
	ggcacgtgtc
	gccatgtggc
	agaaggtggt
	gacacgtggc
	gccacgagga
	tgcacctggt
	accacatgta
	cccacgtggc
	gtcaggtggt
	tacacatggc
	agaacatgga
	gccatgtggc
	ccaacatggt
	tccacatgga
	gccacatgtt
	agcacatgcc
	agcacgtgtc
	acaaagtggc
	cccacgaggc
	agcacgtgat
	tgcaagtggc
	agcacgtggc
	atcacctgga
	ggcacgtgga
	ggaatgtgtt
	aacacgtgag
	ggcacctggc
	cacacgcggc
	aaaacgtgat
	agcacctggc
	ggcgcgtggt
	ggcccgtggc
	gccatgtggt
	ggcacatggc
	atcacgaggt
	tgcacgtgtc
	tgcacgtgtc
	tgcacgtgtc
	acctcgtgac
	aacacttggc
	gacacgcggc
	accatgtgac
	gccaggtgga
	gccacctggt
	acaacgtgga
	agcaagttgt
	agcacatgac
	ggcacctggc
	ggcacgtctt
	accacatgct
	aggccgtggt
	tccacgtgct
	aacacgaggc
	atcacgtgcc

	aaaatgtggt<br>cccacgtgcc<br>gccacgtcga<br>tgtacgtggt<br>gacacgtggt<br>agcagctggt<br>accacgtggg<br>actatgtggt<br>cccaggtggt<br>aggacgtgga<br>ggcaggtggt<br>aaaacatgga
P53	cagggcatctctaaggagga<br>caagaccagcctgggaaaaa<br>ccgggcatgttggtacatgc<br>tggtacatgcctgtagtttc<br>agcagcatgctcaggcatga<br>caggccctgtcttttcatct<br>cgagacaagccaagacatga<br>caagacatgactgggttcat<br>aatgacatgtcctcagtggt<br>tcaaggatgcttggccaggt<br>gctgtcatgtccggggcttg<br>gcacgctagcccaggcatgt<br>gccaccatgcccggctaatt<br>gtgtgcctgccctgggaaca<br>cagaacatgctggagaagag<br>aaaaacatctcctgagctcg<br>ttctgcatgcccggacacgc<br>cgagagaagcctggccaaca<br>gtagacaggctcaggcatgc<br>tcaggcatgccagggttcct<br>aaaggccagctctgggaagg<br>aaggccagctctgggaaggt<br>agagacatgtgtggcagtga<br>tcctgcatgcctgtacttgt<br>gcgcacataccattgcaagg<br>ttaaacatgcctggacaagg<br>ttggccatggccttggaagc<br>gccaacattccaggacgtgc<br>acacacaccccagagcttgt<br>tgtgacaggtcagggcatgc<br>agggtaccgtcctggcatgt<br>accaccacgcctgggctaat<br>tgagaccagcctgggcaaca<br>aggaagttgccctgacaaca<br>ccgggcgtgttggcgcatgt<br>agaggcctgggcggacactt<br>ggtggtgtgcctggacttgt<br>gcgggcgtgtctggaggagc<br>agggacctaccttgagatct<br>tgagagattcgctgccatgt<br>ctgaacaagacagggatttt<br>gccaccatgcccggcctcaa<br>ctagacatcccgaggggtgt
Sp1	gaggcaggga

	ggggcgggat<br>cgggcatggt<br>ggggcaggat<br>gggggagggt<br>cgggcgcggt<br>gggggtggat<br>gggggtggca<br>gtgggaggga<br>gtggcatggt<br>ggggcaggga<br>ggggcaggga<br>tgggcgggat<br>ggggtggggt<br>ggggtggggt<br>gaggttgggt<br>ggggcgtggt<br>gggggggggg<br>gggggggggg<br>gggggggggg<br>ggggggggga<br>ggggcgggga<br>ggggctgggt<br>gtggcgggga<br>tagggtgggt<br>ggggcgggga<br>gaggcggtgt<br>tggggttggt<br>gaggggggt<br>ggggcgggct<br>tgggcttggt<br>tgggcttggt<br>gaggcaggct<br>cgggcaggct<br>gaggctgtgt<br>tgggctgtgt<br>ggggcaggga<br>ggggcaggga<br>tgggcgggat<br>ggggcaggga<br>ggggtggggt<br>ggggtggggt<br>tgggcggggt<br>tgggcgtggt<br>gtgggagggt<br>tgggcggggt<br>ggggctggga<br>gaggcttggt<br>ggggctgggt<br>tgggcagtgt<br>ggggcaggga<br>gaggctggat<br>ctggcagggt<br>gtgggaggga<br>gtggcatggt<br>ggggcaggga<br>ggggcaggga

	tgggcgggat<br>ggggtggggt<br>ggggtggggt<br>gaggttgggt<br>ggggggggga<br>gggggtggca<br>tggggtgggt<br>ggggctggct<br>aaggctgggt<br>tgggtgtggt<br>taggcatggt<br>tgggtgtggt<br>tggggagggt<br>agggcagggt<br>tgggcatggt<br>ggggcaggga<br>ggggcaggga<br>ggggctggga<br>ggggctgggt<br>tgggcagtgt<br>gaggcatggt<br>tgggtggggt<br>ctggcggggt<br>gaggcttggt<br>agggctgggt<br>tgggcagtgt<br>ggggcaggga<br>gtggctggga<br>tagggtgggt<br>tgggcatggt<br>ggggtgtggt<br>gaggctgtgt<br>ggggcaggga<br>ggggcaggga<br>tgggcgggat<br>gaggcatggt<br>gaggcttggt<br>agggctgggt<br>tgggcagtgt<br>ggggcaggga<br>cgggcgtggt<br>gagggtgggt<br>ggggtgggga<br>gaggcgggct<br>ggggcaggga<br>ggggcggtgt<br>aaggcggggt<br>ggggcagggt<br>cgggcgtggt<br>tggggagggt<br>tgggcaggga<br>ggggcggtat<br>ggggcagggt<br>gagggtgggt<br>cagggtgggt<br>gagggaggga

	ggggcggggt<br>tgggcttggt<br>gaggcgggga<br>gaggctctga<br>gaggcaggga<br>gagggtgggt<br>ggggctggga<br>ctggcggggt<br>ggggctgggt<br>gaggcgggga<br>ggggcgctgt<br>ggggcaggat<br>tgggcatggt<br>ggggtggggt<br>ggggggcggt<br>agggcgggga<br>ggggggaggt<br>tgggcgggga<br>gaggcggggt<br>gaggcggggt<br>ggggcgtggt<br>agggctgggt<br>ggggcgtggt<br>agggctgggt<br>tgggcgtggt<br>tgggcatggt<br>aagggtgggt<br>gaggggtgt<br>gggggtgtgt<br>gaggcgtggt<br>tgggcacggt<br>ggggcgggga<br>ggggcggggt<br>ggggcgcggt<br>ggggggcggt<br>ggggcgtggt<br>ggggctgggt<br>ggggcgtggt<br>ggggcgtggt<br>cgggcgtggt<br>ggggcgggga<br>gtggggtggt<br>ggggcgggca<br>tgggtgttgt<br>ttgggtgggt<br>gggggagtgt<br>agggggaggt<br>gagggaggta<br>ggggcattct<br>atgggtggct<br>gaggggtggg<br>aggggtgggc<br>ggggtgggcg<br>agggcagtgt<br>cgggcggggt<br>gaggcttgga

	gaggggcggt<br>agggggggtgt<br>tgggcttggt<br>agggctgtgt
